# The Book World of Medicine and Science

**Published:** 1904-01-09

**Authors:** 


### THE BOOK WORLD OF MEDICINE AND SCIENCE.

THE BRITISH JOURNAL OF CHILDREN'S DISEASES. Edited by GEORGE CARPENTER, M.D. No. 1. Vol. I. January, 1904. (London: Adlard and Sons. Price 1s. net.)

THE first number of this new journal reaches a high level of excellence. There can be no question that in respect to children's diseases there is a distinct gap in British medical journalism and it is satisfactory to find that this defect is to be supplied in a really worthy fashion. If the "British Journal of Children's Diseases" can maintain the standard suggested by its first issue, those who have undertaken the enterprise may rest assured of a well-deserved success. We are glad to notice that the editor appreciates the advantage of publishing in the same number two or more articles on the same subject by different authors. On the present occasion this is illustrated in two instances. Dr. Edmund Cautley and Mr. Clinton Dent write on congenital hypertrophic stenosis of the pylorus, and Dr. G. A. Sutherland and Mr. Harold Burrows contribute each a paper on Henoch's purpura in relation to intussusception. Every one of these articles is of distinct value, whilst the general view of each subject thus presented is greatly improved by the survey from two different standpoints. The discussion of Henoch's purpura is rendered still more complete by an excellent editorial in which the clinical position of this still obscure malady is clearly defined. Another article of great interest is that by Dr. James Taylor on a case of hemicrania associated with paralysis of the third nerve. We shall hope in future numbers to see some contributions in reference to the more common diseases of early life, and with the subject of infant feeding the new journal may be expected to deal in an authoritative fashion. In this direction there is a large opportunity both for destructive criticism and scientific construction. We shall follow the progress of our latest medical contemporary with much interest and with all goodwill.

THE PRINCIPLES AND PRACTICE OF SURGERY. By GEORGE TULLY VAUGHAN. (T. B. Lippincott Co., Philadelphia and London. 569 pages. 281 illustrations. Price 15s. net.)

AT the present time when there are so many and such excellent text-books on surgery one cannot view the publication of a new one with any feelings of joy, and such a one must necessarily be subjected to somewhat severe criticism, and if it does not come up to a high standard it is apt to have only scant attention paid to it. The author in his preface states that the object of this volume is to present the subject of general surgery in the way best adapted to the uses of the general practitioner and student. This is an object much to be commended, but it must be remembered that the general practitioner of to-day is a very highly-trained man and wants the very best, and in our opinion the author has not proceeded in the right manner to give him this. He states that he has left out several branches of special surgery, such as ophthalmology and otology, but he has inserted much that can be of very little practical use to either the practitioner or the student. The opening chapter deals with the subject of inflammation, but treated in a very brief and uncomprehensive manner, so that to the student it would convey little and the practitioner would gain nothing new. In a later chapter quite apart from this one comes another, dealing with inflammation and suppuration in a similar unsatisfactory manner. On reading the book one is much struck by the fact that great attention is paid to the subject of operative surgery, the ligation of vessels and the question of amputations being dealt with at some length. These matters are not of such very great importance to the average student, and as they cannot be dealt with as they deserve in a volume of this capacity they might well, in our opinion, be left out, seeing that there are many excellent books dealing fully with this subject. Even if the author considered this course inadvisable, he might have left out many antiquated and obsolete methods such as Teale's amputation, for instance, which only tend to confuse the student, and which can serve no useful purpose. Again a full description is given of Petit's tourniquet, an instrument probably very few students have ever seen or are likely to see in a modern hospital. A description of the art of bandaging and the giving of anæsthetics, too, take up a considerable amount of space which might have been reserved for better use. Seeing that so much space has been taken up by these matters, it is obvious that the remaining more important surgical ailments and methods of diagnosis and treatment can only receive scant attention, and it is not surprising to see that an important subject, Diseases of the Breast, is dealt with in three and a half pages, the diagnosis of malignant disease of the breast is only briefly referred to, and mastitis and the various forms of cystic disease of the breast, which at this time are exciting so much attention, being passed over without notice. In brief, it may be stated that the book contains a good deal of what is old and very little of any new work, and such being the case it cannot be recommended to either the practitioner or the student. In praise of the volume let it be said that the printing and general get-up are excellent and the illustrations well reproduced, though many of them are, we think, quite unnecessary, and only tend to take up valuable space.

THE PHYSIOGNOMY OF MENTAL DISEASES. By JAMES SHAW, M.D. (Bristol: J. Wright and Co. 1903. Pp. 83. Illustrations 55. Price 3s. net.)

THIS is one of those books of which almost unqualified approval may be expressed. It is small in size; it is clearly put; contains no padding, and we feel sure it will be found alike useful to the specialist and to the general practitioner; but the latter will hardly be able to extract the full benefit from it unless he can make a careful study of a few types of insanity in the asylum of his county. With this book in his hand and an asylum medical officer at his elbow much might be learned in a very short time. The title would lead one to suppose that the scope of the book is more limited than it really is. Scattered throughout are remarks of diagnostic value. For instance, when describing acute delirium the author points out the importance of its differential diagnosis from acute mania and agitated melancholia; and he says it may be distinguished from the former by the help of such non-facial signs as temperature, as well as by the total absence of facial reaction, and by the tremor, and from the latter by the facial colouring and by the absence of persistent expression of acute mental pain. Again, in epileptic mania it is stated that during the paroxysms or attacks "the facial expression denotes anger and suspicion, and, as a rule, there is either no facial reaction or one indicating an outburst of rage." Most of the letterpress has already appeared in serial publications, and assuredly the author has chosen wisely in presenting it in book form, giving us almost at a glance a section of mental medicine hitherto not easily accessible. The illustrations are decidedly good and vividly recall to mind the various types of insanity.

APPOINTMENTS. 1904. (London: Printed by Charles Letts and Co.)

WE have received from Messrs. Dargue, Griffiths and Co., the well-known warming and ventilating engineers, a very neat and handy little appointment book for 1904. In addition to containing sufficient space to note down engagements for every day in the year, there are extra pages for ordinary memoranda and for a cash account. Some useful postal and other information is also given.

